# Supplementary material for: Efficacy of short-course treatment for prevention of congenital transmission of Chagas disease: A retrospective cohort study
Source: PLoS Negl Trop Dis. 2024 Jan 22;18(1):e0011895. doi: 10.1371/journal.pntd.0011895 (PMC10833510; doi:10.1371/journal.pntd.0011895)
Supplement: Attachment — Submitted filename: Answer letter to editor.docx [file pntd.0011895.s001.docx]

Buenos Aires, November 29^th^, 2023

Richard Reithinger,

Academic Editor, PLOS NTD

Dear Richard Reithinger,

**Re: Efficacy of short-course treatment for prevention of congenital transmission of Chagas disease: a retrospective cohort study. (PNTD-D-23-01178)**

We have carefully reviewed the comments on our manuscript: “Efficacy of short-course treatment for prevention of congenital transmission of Chagas disease: a retrospective cohort study”.

We found the comments very useful. We believe we have fully answered your comments and concerns, and are re-submitting for your consideration. Please find the specific answers below.

Kind regards,

Dr. Guillermo Moscatelli

Parasitology and Chagas Service,

Buenos Aires Children’s Hospital “Dr. Ricardo Gutiérrez”

Gallo 1330, (1425) Buenos Aires, Argentina

Tel: +5411-4963-4122

Email: gfmoscatelli@gmail.com

**Specific answers to Reviewers ‘comments:**

| **Reviewer #1** | | |
| --- | --- | --- |
| **We are grateful for your suggestions that have allowed us to improve our work** | | |
| **Methods** | **Comments** | **Answer** |
|  | Objectives were clearly articulated, and the study design was appropriate.  - What ELISA did you use? Lysate or recombinant? | The ELISA used for our study was lysate |
|  | Related to the Ethics clearance, can you provide the study number or ref? | The reference number of the study is CEI 16.29. |
|  | Sample size is limited. This is a limitation of the study. | Although the sample size is limited, there was no transmission of congenital Chagas disease in any case, coinciding with previous reports that used treatment for 60 days. We intend to continue to include patients to confirm these preliminary findings. |
|  | No statistical analysis was included. For instance, were statistically significant the variations in the ELISA reactivity pre- and post-treatment? | Statistical analysis is included in methods and results. A comparative figure of anti-T. cruzi antibody survival is now included also added in the results. |
| **Results** | The fact that two of the analyzed participants indeed cured the infection is a very interesting observation emphasizing the efficacy of treatment. However, for the purposes of the study these two mothers shouldn´t have been included as being negative to *T. cruzi* infection at the time of delivery, meaning there was no risk of transmission. | Regarding the two patients who tested negative for conventional serology, we considered them included because they met the inclusion criteria (Children of mothers who received treatment with BZ or NF for less than or equal to 30 days). The aim of the study is precisely to demonstrate the efficacy of the treatment in preventing congenital infection, and secondly to show its efficacy in curing the disease. In fact, negativisation of parasitological methods and a sustained decrease in conventional serology are also markers of therapeutic response, indicating the efficacy of treatment of the disease. |
|  | In terms of the PCR results of the children, would it be possible to have a figure showing these? | In relation to plotting the PCR results in children, we consider it is not representative, since all results were non-detectable. |
|  | It should depict age and include the Ct values (mean and SD) of the reactions? Same should be made for the women PCR results if they come from qPCR amplification protocol. | The Limit of Detection (LOD) of T. cruzi DNA qPCR is 0.70 par. eq./mL (95%CI, 0.54-1.01 par.eq./mL) (Ramirez et al., 10.1016/j.jmoldx.2015.04.010). All qPCRs were perfomed in uniplicate. |
|  | Regarding the serological results of the children, although a negative general result was obtained, could you please include a figure showing it? If possible, it should include the OD values of positive controls for comparison. | The figure of PCR results in mothers would also not be representative, as all women had negative PCR results at the end of treatment. Regarding children serology, a serological curve was not conducted before 8 months of age due to the passive transfer of maternal antibodies. A follow-up study of children born to mothers with Chagas is currently underway, where we will be able to assess the differences between infected and non-infected individuals in their serological studies (ClinicalTrials.gov NCT´04084379). |
|  | Minor:  - Line 207: "... cases have been ..." change for "... there are ...". | We have corrected the suggestion on line 207 |
| **Conclusions** | The low transmission rate of vertical T. cruzi infection poses a major limitation to the study due to the small cohort size of women who received treatment before delivery. If an average 5% vertical transmission rate is considered only 2 children out of the 40 would have been infected. Please can you comment about this in the Discussion? | We have included in the discussion the suggestion to limit the number of incorporated patients. |
|  | Statement in lines 235 and 236: can you further clarify if that >20% seroreduction is a recommendation of WHO-PAHO? | The CHICO-SECURE study, which allowed nifurtimox to be registered with the FDA, used this therapeutic response criterion. These criteria were validated in a study involving a large cohort of patients treated by our group. This information is added to the discussion. |
|  | In relation to the two cases that showed cure (subject mothers 1 and 2), if they were negative at the time of considering them for the study, their newborns should not have been included. As stated before, it is a great result to ensure that treatment worked, but their children were then born to non-infected women. | We believe that the two patients who tested negative for conventional serology should be included because the study includes women with Chagas disease treated prior to pregnancy, regardless of their disease status. The aim of the study is to demonstrate the efficacy of treatment in preventing congenital infection and, secondarily, to show the efficacy of treatment in curing the disease. |
|  | Regarding the treatment evaluation, optimism is highly appreciated, but a more realistic message in relation to the timely evaluation of treatment response is missing. Lack of bmks for the early assessment of therapeutic efficacy is a major drawback for the follow-up of subjects who recieved anti-parasitic drugs, and sure having to wait for 36 months to address it is not practical at all. | This study confirms previous data using shorter treatment times and using as an outcome a clinical event of non-transmission of infection. |
|  | In line 244 you mention "sustained decline" in the serological outcome of treated women, but there are 3/27 (or 3/25) whose antibody evolution reflected an increase by ELISA, which is considered to have a better sensitivity than IHA. Maybe it would be better to reword that sentence, suggesting that a majority of the women treated experienced a decline in the Ab reactivity by ELISA and IHA when evaluated 36 months after administration. But, according to current guidelines, none was really cured except for the first two girls. A short duration of treatment was effective in preventing vertical transmission but whether the women are cured would require further evidences. | This study is pragmatic within a real healthcare setting where we show antibody drop and negativisation. |
|  | What about shortened administration regimes for the infected newborns. Generally, they much better tolerate benznidazole and nifurtimox than adults, but yet it would be greatly beneficial to have shorter administration times. Can you provide a few lines on this matter? | Since all infants were not infected, they did not receive treatment. Line 228 shows the first prospective clinical trial in children with Chagas disease, comparing 30-day and 60-day NF regimens. It showed similar efficacy results with both regimens, especially in children under 2 years of age. |
| **Editorial and Data Presentation Modifications** | Please use italics for species names like *Trypanosoma cruzi*. | The typography has been corrected |
| **Summary and General Comments** | The work presents a retrospective cohort study of *T. cruzi*-infected women who received halved anti-parasitic treatment and evaluates whether this sufficed to avoid vertical transmission of the infection to their offspring. Results are interesting and this kind of studies is greatly needed by the Chagas community. However, it is limited in size, and a more detailed analysis of the diagnostic results should be provided to better support the treatment response statements. In its current shape it might fit better as short report than as an original article format. | These suggestions have been modified in the text on the basis of each comment. |

| **Reviewer #2** | | |
| --- | --- | --- |
| **We are grateful for your suggestions that have allowed us to improve our work** | | |
| **Methods** | **Comments** | **Answer** |
|  | The objective of this study is clear and has a logical line and clearly articulated.  The design is consistent with the objective. And the study design is satisfactorily developed.  Line 110: There is an error in the inclusion criterion: children of mothers treated for at least 30 days with etiological treatment for *T. cruzi* infection. Instead it should say less than or equal to 30 days of treatment, to be consistent with the objective of the study. | The error in the inclusion criteria has been corrected. |
|  | Being a cohort study, of an observational nature in a specific period of time, the power of the evidence is not evaluated because there are only 27 cases involved. | Although the sample size is limited, there was no transmission of congenital Chagas disease in any case, coinciding with previous reports that used treatment for 60 days. We intend to continue to include patients to confirm these preliminary findings. |
| **Results** | The data is clear and is shared correctly, as shown in Table 1. Although I recommend including a table where it can be easier to understand the accumulated values, according to type of treatment, days of treatment, age ranges of having received treatment, and other priorities variables in Table 1, although with accumulated data. | The table was organized according to days of treatment and the cumulative data are described in the text. |
| **Conclusions** | The conclusions are supported by the data presented, however it is perceived that the limitations of the type of study and analysis are not clearly described in the discussion. It is important that the authors can propose that more evidence is necessary in this same approach in order to change and/or adapt the recommendations related to the duration of treatment. | One limitation of our study is the number of patients included, however there was no transmission of congenital Chagas disease in any case, coinciding with previous reports that used treatment for 60 days. Further studies are necessary in order to confirm these preliminary findings. This statement is added to the discussion. |
| **Editorial and Data Presentation Modifications** | Line 80, remove stitch after Nifurtimox (NF).  Line 87, translate ETMI-plus: Elimination of mother-to-child transmission (EMTCT)-plus  Line 107: Change and/or include, in addition to Chagas Disease, *T. cruzi* infection, because the majority of those treated were not sick, they just had the infection. It is also recommended to make this change throughout the entire text where this type of expression is found.  Line 110: There is an error in the inclusion criteria: children of mothers treated for at least 30 days with etiological treatment for *T. cruzi* infection. Instead it should say less than or equal to 30 days of treatment, to be consistent with the objective of the study.  Line 115: While it is said that the follow-up time with serology is at least 3 years, in relation to parasitological tests (real-time polymerase chain reaction - *T. cruzi* qPCR) it says before and after without precise time, it would be important to include this information.  Lines 199 -203: It seems important to include that the evidence generated by studies on the effectiveness of etiological treatment in girls and women of childbearing age to avoid congenital transmission, has been key for its inclusion in regulations/guidelines for diagnosis and treatment for Chagas from both the PAHO as from endemic countries. | We have modified the suggestions on lines 80, 87, 110. In relation to line 107 we believe that the definition is correct since most patients with Chagas disease are asymptomatic throughout their lives. Line 115 is corrected by clarifying the time of PCR, being at the end of treatment. The comments suggested in lines 199-203 have been added. |
| **Summary and General Comments** | This manuscript is interesting and important for the neglected diseases and public health community because it contributes to reviewing the evidence on simplifying care procedures and thus improving and expanding access to antiparasitic treatment for Chagas. Furthermore, the relevance of this manuscript is due to its contribution to a specific population, pointing to a possible health policy that considers the elimination of congenital transmission. I recommend for your better understanding and discussion that the suggestions made in the review be included. | Thank you for highlighting the importance of our manuscript. We aimed to contribute valuable insights into simplifying Chagas treatment procedures, enhancing access to antiparasitic therapies, and addressing congenital transmission. Your suggestion to include the review's suggestions for a more comprehensive discussion is appreciated and will be considered during revisions. We are committed to refining our work for greater impact. |
